# Scientific, bibliometric and biographical analysis of 71 Jewish and dissident pharmacologists persecuted in Germany between 1933 and 1945

**DOI:** 10.1007/s00210-024-03645-z

**Published:** 2024-12-18

**Authors:** Mirja Mispagel, Roland Seifert

**Affiliations:** Institute of Pharmacology, Hannover Medical School, Carl-Neuberg-Str. 1, D-30625 Hannover, Germany

**Keywords:** National socialism, *Naunyn–Schmiedeberg’s Archive of Pharmacology*, Persecuted pharmacologists, Publication activity

## Abstract

*Naunyn–Schmiedeberg’s Archives of Pharmacology*, founded in 1873, is the oldest pharmacological journal. This study sheds light on the influence of persecution and expulsion of Jewish and dissident German pharmacologists during the Nazi era (1933–1945) on their scientific work and publication behaviour. The analysis is based on the German-language book ‘*Verfolgte deutschsprachige Pharmakologen* (persecuted German-speaking pharmacologists) *1933–1945*’ by Trendelenburg and Löffelholz (2008), which contains short biographies of 71 persecuted pharmacologists. We analysed their publication activity from 1900 to 1980, the topics of the publications and the emigration data. Most persecuted pharmacologists emigrated, with two peaks of emigration around 1933 and 1938. Most pharmacologists emigrated to the USA, followed by Great Britain. Five of the scientists who emigrated to Great Britain were elected to the *British Pharmacological Society’s Pharmacology Hall of Fame*, and one of them was a Nobel Laureate. Very few of the emigrated pharmacologists returned to Germany. After the Nazis came to power in 1933, the share of papers by persecuted pharmacologists in *Naunyn–Schmiedeberg’s Archives of Pharmacology* dropped sharply. At around 1936, several of the persecuted pharmacologists began to publish increasingly in the American competitor journal, the *Journal of Pharmacology and Experimental Therapeutics*. The persecuted pharmacologists who emigrated to Great Britain had a major influence on the *British Journal of Pharmacology*, founded in 1946, as initially, they accounted for a high proportion of publications. We further analysed the papers published in *Naunyn–Schmiedeberg’s Archives of Pharmacology* by persecuted pharmacologists between 1933 and 1945. About half of these papers were submitted from abroad, indicating that despite the persecution and repression, papers from persecuted pharmacologists previously working at German institutes were still published during this period. Most of the papers by persecuted pharmacologists published from German institutes during this period were published under regime-critical or politically persecuted institute directors. Persecuted pharmacologists covered a huge spectrum of scientific topics, highlighting their immense scientific impact. After World War II, *Naunyn–Schmiedeberg’s Archives of Pharmacology* lost much of its previous thematic diversity for decades. Overall, our analyses highlight the enormous loss to German pharmacology due to the persecution, exclusion and expulsion of ‘non-Aryan’ pharmacologists. Conversely, pharmacology of the USA and Great Britain benefited greatly from the emigration of distinguished scientists from Germany.

## Introduction

*Naunyn–Schmiedeberg’s Archives of Pharmacology* is the oldest pharmacological journal (Starke 1998) and had its 150th anniversary in 2023 (Hattori and Seifert 2023; Dats et al. 2023). On this anniversary, several historic articles have been published so far, covering the founders of pharmacology, Rudolf Buchheim (Toomsalu 2023) and Oswald Schmiedeberg (Greim 2024a), the schools of pharmacology at the universities of Tartu (Philippu and Seifert 2023a) and Strasbourg (Philippu and Seifert 2023b), the overall bibliometric development of *Naunyn–Schmiedeberg’s Archives of Pharmacology* (Dats et al. 2023), the contribution of German pharmacologists to the development of pharmacology in Japan (Hattori et al. 2023), the development of pharmacology and toxicology in Munich (Eyer 2024; Greim 2024b), and the development of pharmacology in Germany after World War II, with particular emphasis on the differences between East- and West-Germany and East- and West-Berlin (Basol and Seifert 2024a, b).

The period of the Nazi regime in Germany from 1933 to 1945 was a dark time for science and medicine, including pharmacology. Jewish pharmacologists, like many other Jewish and dissident scientists, faced persecution, marginalisation and violence under this regime (Medawar and Pyke 2001). In a previous paper, Löffelholz (2011) described the fate of some prominent German and Austrian pharmacologists during the Nazi regime and honoured them. This study is a continuation of the work by Löffelholz (2011) in the context of historical articles appearing on the 150th anniversary of the journal.

Here, we analysed the far-reaching consequences of the persecution of German-speaking pharmacologists, examining their biographies and publications in the German pharmacological journal *Naunyn–Schmiedeberg’s Archives of Pharmacology,* the *British* *Journal* *of* *Pharmacology* and the American *Journal of Pharmacology and Experimental Therapeutics*. This study was based on the book ‘*Verfolgte deutschsprachige Pharmakologen* (Persecuted German-speaking pharmacologists) *1933–1945*’ by K. Löffelholz and U. Trendelenburg (2008). In particular, the selection of the 71 persecuted pharmacologists analysed here is based on that book. To our knowledge, this is the first systematic presentation of German pharmacologists persecuted during the Nazi era in English language. In addition, this paper analyses the research topics of the persecuted pharmacologists between 1900 and 1980. In a study that is underway, we will analyse in detail the individual fates of persecuted pharmacologists. The present paper focuses on a global scientific, bibliometric and biographical analysis of persecuted pharmacologists.

Our study shows how aggressive political ideologies can have a far-reaching and long-lasting impact on impeding scientific freedom and progress. Considering current political developments, which show a worrying shift to nationalism and populism-based political parties or movements, this study is highly relevant (Ashe et al. 2020). Constant reappraisal is important in order to keep the historical events and their consequences of National Socialism present within society.

## Materials and methods

### Selection of pharmacologists

The book ‘*Verfolgte deutschsprachige Pharmakologen 1933–1945*’ by K. Löffelholz and U. Trendelenburg (Löffelholz and Trendelenburg 2008) served as the basis for our research on persecuted pharmacologists in Germany during the Nazi era. This book contains short biographies of 71 pharmacologists who were persecuted during the Nazi era for their Jewish origin or for being politically dissident (Table 1). We have taken from the book the dates of birth and death, as well as the individual fates of the 71 pharmacologists. In the case of emigration, we also took the year and country of emigration from the book. It is well possible that more than the 71 pharmacologists documented by Löffelholz and Trendelenburg (2008) were persecuted during the Nazi era, but we do not have appropriate data to document any potentially missing individuals.

**Table 1:** Key biographical data of 71 pharmacologists persecuted during the Nazi era

	Persecuted pharmacologist (family name, given name)	Year of birth	Year of death	Gender	Degree programmes	Destiny	Emigration year	Emigration to
1	Bergmann, Felix Eliezer	1908	2002	m	Medicine, chemistry	Emigration	1933	Palestine
2	Blaschko, Hermann Karl Felix	1900	1993	m	Medicine	Emigration	1933	GB
3	Born, Gustav Victor Rudolf	1921	2018	m	Medicine	Emigration	1933	GB
4	Brauer, Ralph Werner	1921	2000	m	Chemistry, biochemistry	Emigration	1937	USA
5	Bueding, Ernst B. (alias Bueding, Ernest B.)	1910	1986	m	Medicine	Emigration	1933	USA
6	Bülbring, Edith	1903	1990	f	Medicine	Emigration	1933	GB
7	Copley, Alfred Lewin	1910	1992	m	Medicine	Emigration	1935	USA
8	Dresel, Peter	1925	1987	m	Chemistry, biology	Emigration	1938	USA
9	Ellinger, Friedrich P.	1900	1962	m	Medicine	Emigration	1936	USA
10	Ellinger, Philipp	1887	1952	m	Medicine, chemistry	Emigration	1933	GB
11	Feldberg, Wilhelm Siegmund	1900	1993	m	Medicine	Emigration	1933	GB
12	Forst, August Wilhelm	1890	1981	m	Medicine, chemistry	Stayed in Germany	/	
13	Freund, Ernst	1863	1946	m	Medicine, chemistry	Emigration	1938	GB
14	Freund, Hermann	1882	1944	m	Medicine, chemistry	Murdered in Concentration Camp (1944)	1940	Netherlands
15	Fröhlich, Alfred	1871	1953	m	Medicine	Emigration	1939	USA
16	Glaubach, Susi	1893	1964	f	Chemistry	Emigration	1938	USA
17	Grab, Werner	1903	1965	m	Medicine	Stayed in Germany	/	
18	Griesbach, Walter Edwin	1888	1968	m	Medicine	Emigration	1939	New Zealand
19	Handovsky, Hans	1888	1959	m	Chemistry	Emigration	1933	Belgium
20	Hausmann, Walther	1877	1938	m	Medicine	Suicide (1938)	/	
21	Havemann, Robert	1910	1982	m	Chemistry	Stayed in Germany	/	
22	Heller, Hans Sigmund	1905	1974	m	Chemistry	Emigration	1934	GB
23	Hellmann, Kurt	1922	2013	m	Chemistry	Emigration	1933	GB
24	Henze, Carlo	1907	2003	m	Medicine	Emigration	1938/39	USA
25	Herxheimer, Andrew	1925	2016	m	Medicine	Emigration	1938	GB
26	Herxheimer, Herbert Gotthold Joachim	1894	1985	m	Medicine	Emigration	1938	GB
27	Holz, Siegbert	1911	1999	m	Medicine	Emigration	1933	Venezuela
28	Jacoby, Martin Johann	1872	1941	m	Medicine	Emigration	1939	GB
29	Kapeller-Adler, Regina	1900	1991	f	Chemistry	Emigration	1939	GB
30	Kochmann, Martin	1878	1936	m	Medicine	Suicide in Prison (1936)	/	
31	Kohn, Richard (alias Richards, Richard Kohn)	1904	1983	m	Medicine	Emigration	1935	USA
32	Kosterlitz, Hans	1903	1996	m	Medicine	Emigration	1933	GB
33	Krayer, Otto	1899	1982	m	Medicine	Emigration	1933	USA
34	Laqueur, Ernst	1880	1947	m	Medicine, chemistry	Stayed in Germany		
35	Lehr, David	1910	2010	m	Medicine	Emigration	1938	USA
36	Lipschitz, Werner Ludwig	1892	1948	m	Medicine, chemistry	Emigration	1933	USA
37	Loewe, Siegfried Walter	1884	1963	m	Medicine	Emigration	1933	USA
38	Loewi, Otto	1873	1961	m	Medicine, chemistry	Emigration	1938/39	USA
39	Maengwyn-Davies, Gertrude Diane	1910	1985	f	Pharmacy, chemistry	Emigration	1938	USA
40	Marquardt, Peter	1910	1997	m	Medicine, chemistry	Stayed in Germany		
41	Mautner, Hans	1886	1963	m	Medicine	Emigration	1938	USA
42	Mautner, Henry G	1925	1995	m	Chemistry	Emigration	1938	USA
43	Meier, Rolf	1897	1966	m	Medicine	Emigration	1935	Switzerland
44	Meyer, Hans Horst	1853	1939	m	Medicine, chemistry	Stayed in Germany	/	
45	Molitor, Hans	1895	1970	m	Pharmacy, medicine	Emigration	1932	USA
46	Müller, Franz	1871	1945	m	Medicine, chemistry	Emigration	1935/37	Brazil
47	Noether, Paul	1888	1933	m	Chemistry	Suicide (1933)	/	
48	Oppenheimer, Ernst	1888	1962	m	Medicine	Emigration	1936	USA
49	Peters, Georg (alias Peters, Georges)	1920	2006	m	Medicine, biochemistry	Emigration	1937/38	Turkey
50	Pick, Ernst Peter	1872	1960	m	Medicine	Emigration	1938	USA
51	Pietrkowski, Georg (alias Peters, George)	1874	1964	m	Medicine	Emigration	1933	USA
52	Pollak, Leo	1878	1946	m	Medicine	Emigration	1939	GB
53	Pulewka, Paul	1896	1989	m	Medicine	Emigration	1935	Turkey
54	Riesser, Otto	1882	1949	m	Medicine, chemistry	Emigration	1939	Netherlands
55	Rosenberg, Walter (alias Rudolf Vrba)	1924	2006	m	Chemistry, biochemistry	Emigration	1944	Slowakia
56	Schild, Heinz Otto	1906	1984	m	Medicine	Emigration	1932	GB
57	Schlesinger, Max (alias Slazenger, Max)	1903	1971	m	Medicine, political science	Emigration	1933	Burma/Myanmar
58	Schlossmann, Hans	1894	1956	m	Medicine	Emigration	1935	GB
59	Schnitzer, Robert Julius	1894	1987	m	Medicine	Emigration	1939	Canada
60	Slotta, Karl Heinrich	1895	1987	m	Chemistry	Emigration	1935	USA
61	Starkenstein, Emil	1884	1942	m	Medicine	Murdered in Concentration Camp (1942)	1939	Netherlands
62	Sulman, Felix Gad	1907	1986	m	Veterinary medicine, human medicine	Emigration	1933	Palestine
63	Taubmann, Gert	1900	1983	m	Medicine	Stayed in Germany	/	
64	Unna, Klaus Robert Walter	1908	1987	m	Medicine	Emigration	1933	USA
65	Vogt, Marthe Louise	1903	2003	f	Medicine, chemistry	Emigration	1935	GB
66	Waelsch, Heinrich Benedict (alias Waelsh, Heinrich Benedict)	1905	1966	m	Medicine	Emigration	1938	USA
67	Wasicky, Richard Balthasar	1884	1970	m	Medicine, pharmacy	Emigration	1938	Brazil
68	Wilbrandt, Walther	1907	1979	m	Medicine	Emigration	1934	Switzerland
69	Wolff, Paul O	1894	1957	m	Medicine	Emigration	1933	Switzerland
70	Wollenberger, Albert	1912	2000	m	Medicine, biology	Emigration	1933	USA
71	Zak, Emil Rudolf	1877	1949	m	Medicine	Emigration	1939	USA

Data in this table were extracted from the German-language book of Löffelholz and Trendelenburg (2008) on persecuted German pharmacologists. The pharmacologists tabulated here constitute the basis for the analyses performed

*m* male, *f* female, *GB*, Great Britain, *USA*, United States of America

### Metadata biographies

We analysed the gender distribution of the pharmacologists included in this study, as well as the degree programmes they completed. We also analysed the countries to which the persecuted pharmacologists emigrated and the years of their emigration. We defined the country of emigration as the country in which the pharmacologist spent most of his or her life and scientific career. Any previous destination countries with shorter periods of residence were not included. We divided the pharmacologists’ fates into five different categories: ‘Emigration’, ‘Remained in Germany’, ‘Returned to Germany’, ‘Suicide’ and ‘Murdered’.

### Metadata on publications—analysis of publications in *Naunyn–Schmiedeberg’s Archives of Pharmacology*, the *Journal of Pharmacology and Experimental Therapeutics* and the *British Journal of Pharmacology*

*Naunyn–Schmiedeberg’s Archives of Pharmacology*, founded in 1873, is the oldest pharmacological journal (Starke 1998; Dats et al. 2023). The complete digital archive of *Naunyn–Schmiedeberg's Archives of Pharmacology* is accessible via SpringerLink (https://link.springer.com/journal/210, last accessed 6 October 2024). Since social and biographical aspects were also important in our study, for example as revealed by congress contributions, we included all contributions published in *Naunyn–Schmiedeberg’s Archives of Pharmacology* by the 71 persecuted German pharmacologists. We collated the relevant data and created charts using Microsoft Excel. The extracted information included bibliometric details such as authors’ names, article titles, affiliations, submission and publication dates, volume numbers, number of citations and the Digital Object Identifier (DOI).

To investigate whether and to what extent emigrated pharmacologists published in alternative journals after emigration, we selected two probable target journals for comparative analyses. As the USA and Great Britain were the most common destination for persecuted pharmacologists, we selected the *Journal of Pharmacology and Experimental Therapeutics* (JPET) and the *British Journal of Pharmacology* (BJP) for comparative analyses. The *Journal of Pharmacology and Experimental Therapeutics* was established in 1909 and, like *Naunyn–Schmiedeberg’s Archives of Pharmacology*, is one of the oldest existing journals in the field, published by the *American Society for Pharmacology and Experimental Therapeutics* (ASPET). The *Journal of Pharmacology and Experimental Therapeutics* also provides an online archive (https://jpet.aspetjournals.org/content/by/year, last accessed 6 October 2024). The *British Journal of Pharmacology*, published by the *British Pharmacological Society* was established in 1946. The full archive of the *British Journal of Pharmacology* is accessible online at https://bpspubs.onlinelibrary.wiley.com/journal/14765381 (last accessed 11 November 2024). Using the author search, we documented all papers published there by the persecuted pharmacologists, extracted other relevant data and created visual representations using Microsoft Excel.

### Metadata topics—analysis of the most common topics using *Basic Knowledge of Pharmacology*

We analysed the topics of published articles from 1900–1980 using the pharmacology textbook ‘*Basic Knowledge of Pharmacology*’ (Seifert 2019) to perform a systematic categorisation, assigning each article to one book chapter. This analysis allowed comparison with contemporary topics covered in *Naunyn–Schmiedeberg’s Archives of Pharmacology*.

We added the topics ‘Poisons and Intoxications’ and ‘Hypothalamic–Pituitary–Adrenal Axis’, which are not originally chapters in the textbook, but are common topics in the articles analysed. Articles that could not be assigned to any of the defined main topics were grouped together as 'Other'. We analysed the changes in research topics over the decades (1900, 1910, etc.) and used Excel to create graphs. This categorisation made it possible to highlight topic developments over the defined period and make comparisons with the general development of topics covered in *Naunyn–Schmiedeberg’s Archives of Pharmacology* (Dats et al. 2023; Basol and Seifert 2024a,b).

### Metadata of articles published between 1933 and 1945

We analysed the countries from which the papers authored by persecuted pharmacologists during the Nazi regime between 1933 and 1945 were published. We also identified the heads of institutes, during whose directorship the papers published from Germany appeared, and analysed their political background. We considered whether the heads of institutes were members of a party or organisation such as the NSDAP, SS or SA, or whether they belonged to a politically persecuted group.

## Results and discussion

### Metadata biographies

#### Gender distribution and degree programmes

**Fig. 1:**
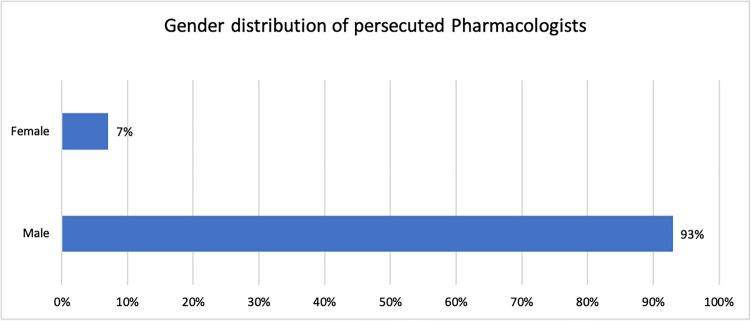
Gender distribution of persecuted pharmacologists

**Fig. 2:**
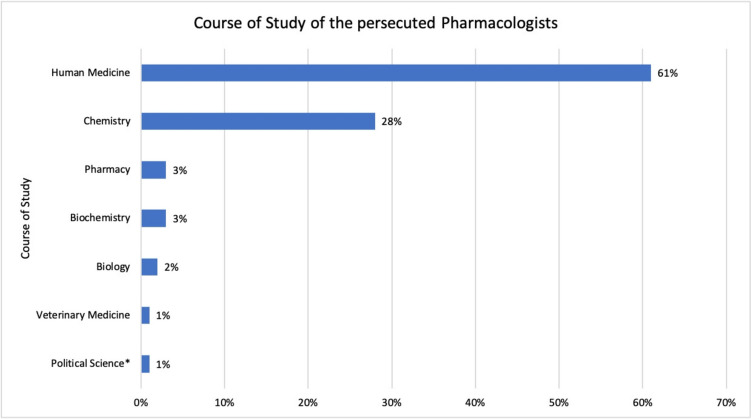
Degree programmes of the persecuted pharmacologists. *The ‘Political Science’ degree programme was an additional qualification to the ‘Human Medicine’ degree programme (see **57** in Table 1)

An analysis of the gender distribution of the persecuted pharmacologists revealed that the vast majority were men, who made up 93% of the group, while women accounted for only 7% (Table 1 and Fig. 1) (**6, 16, 29, 39, 65** in Table 1). We identified the study programmes of the pharmacologists included in our study using the short biographies from the book ‘*Verfolgte deutschsprachige Pharmakologen 1933–1945*’ by Löffelholz and Trendelenburg (Löffelholz and Trendelenburg 2008) (see Fig. 2). Most pharmacologists studied human medicine (61%), followed by chemistry (28%). 3% of the pharmacologists studied pharmacy and/or biochemistry and 2% biology. Veterinary medicine and political science were the least represented fields of study, with 1% each. 32% of the persecuted pharmacologists completed two degree programmes, highlighting the excellent academic education of this group.

#### Emigration and fate

The majority (83%) of persecuted pharmacologists emigrated abroad. A smaller proportion (10%) remained in Germany, 4% of the pharmacologists committed suicide, and 3% were murdered in concentration camps. Table 1 summarizes the emigration status of all the 71 persecuted pharmacologists. The suicide rate of 4% among the pharmacologists analysed here roughly agrees with the range of the general suicide rate of Jews during the National Socialist era in Germany, which was 1–4%, even if the corresponding data could only be approximated (Kwiet 1984). The suicide rate increased dramatically in the years after the Nazis came to power, and waves of suicides could be correlated with historical events such as the ‘Boycott of Jewish Businesses’ of April 1933, the Kristallnacht of 1938, and the deportations of 1942/43 (Kwiet 1984; Duckwitz and Groß, 2021).

**Fig. 3:**
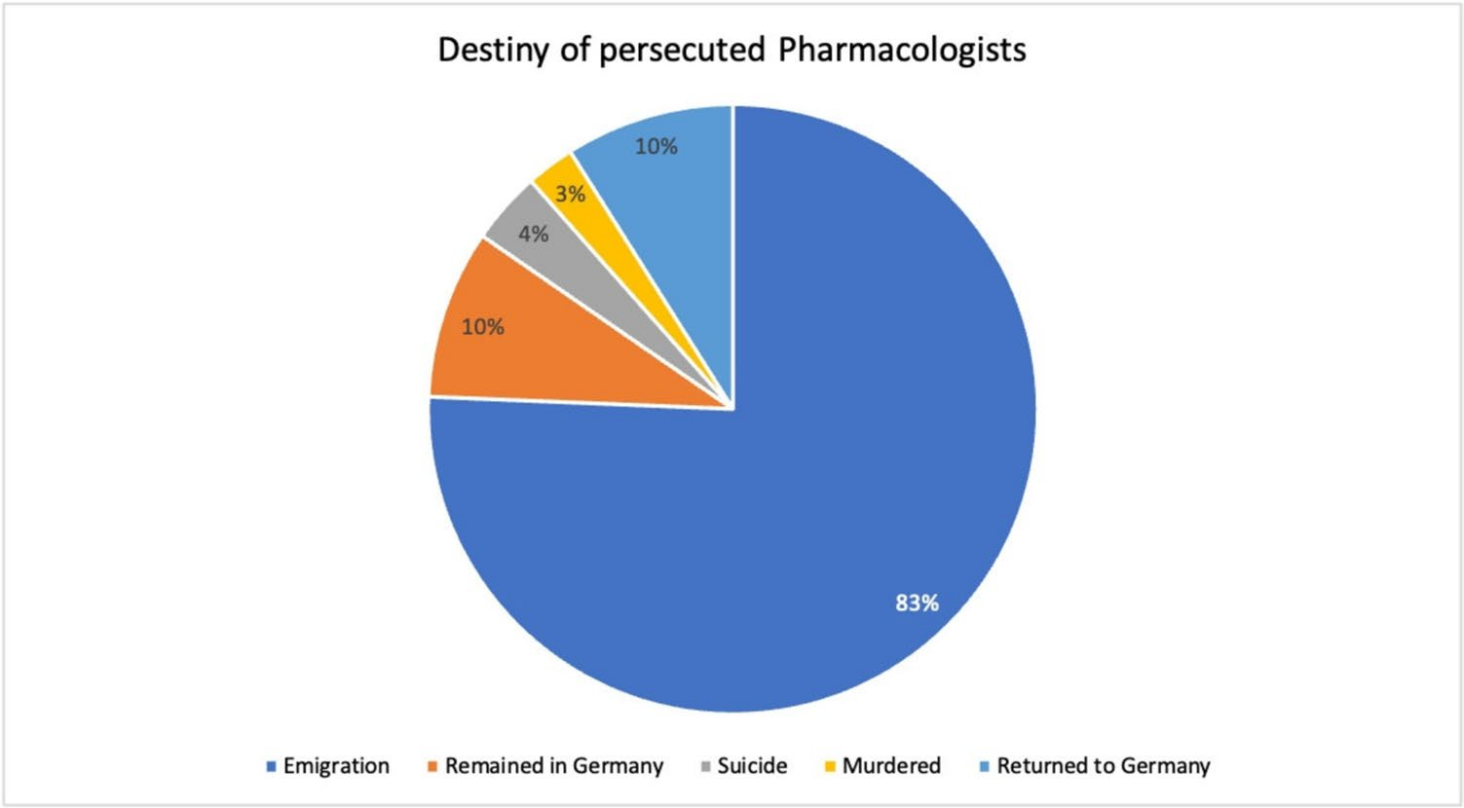
Fate of persecuted pharmacologists

Of the 10% of pharmacologists who emigrated, only a small number returned to Germany after emigration and the end of the Second World War (see Fig. 3). The low rate of returnees to Germany after emigration shows the strong break that the scientists experienced with their former home country as a result of persecution, defamation and expulsion. The break with the country in which they had previously played an important and significant role in the previously highly successful German scientific community was radical (Medawar and Pyke 2001; Rall 2017). Some of the scientists also found the ‘less authoritarian, more informal intellectual atmosphere in Great Britain and the United States much more congenial and receptive to new ideas’ (Medawar and Pyke 2001, p. 47).

#### Years of emigration

**Fig. 4:**
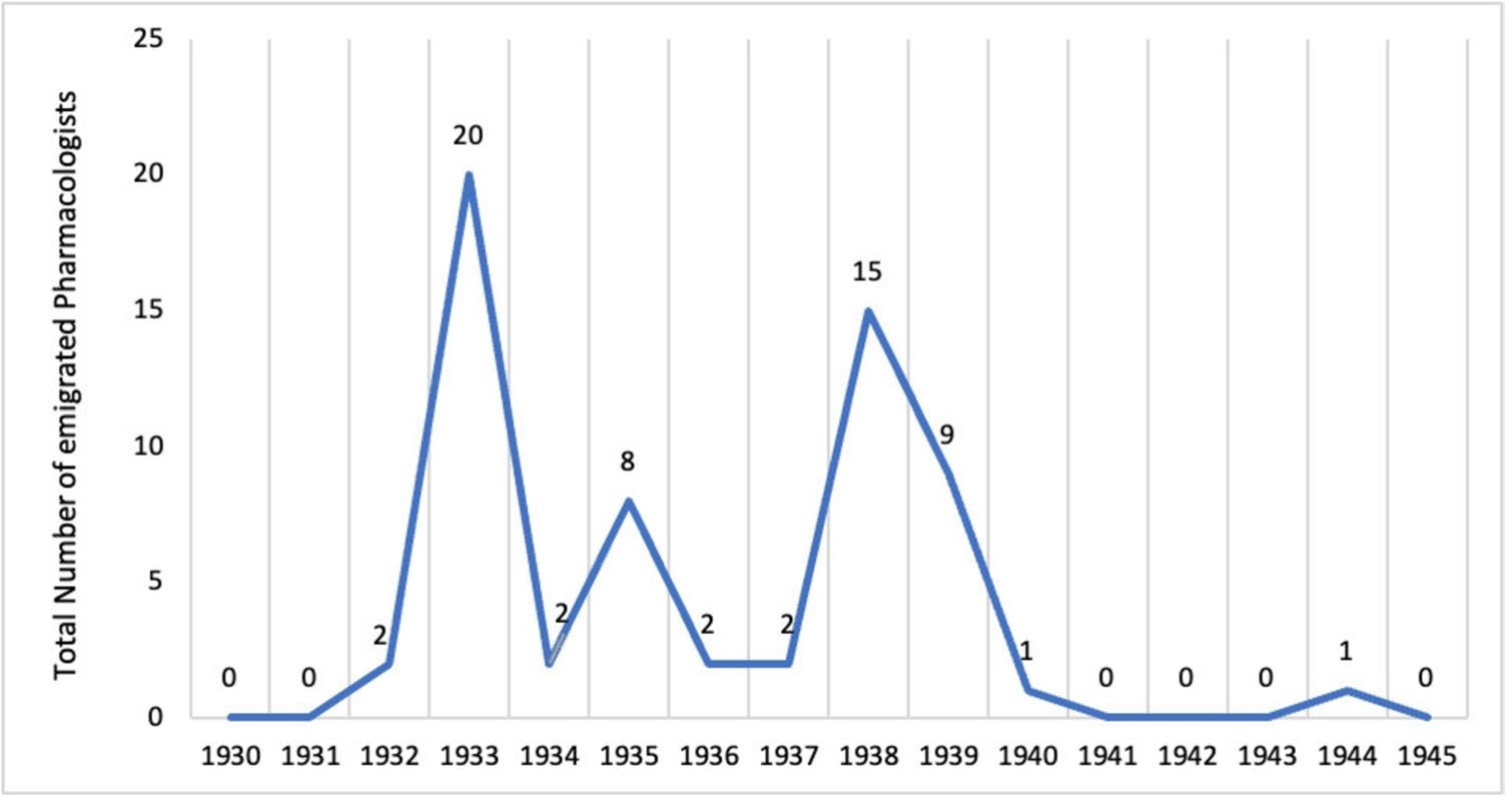
Waves of emigration of persecuted pharmacologists between 1930 and 1945

We analysed the years in which pharmacologists left Germany. Two peaks emerge: 33% of pharmacologists emigrated in 1933 and 25% in 1938 (see Fig. 4). A very similar pattern emerged in a study investigating the persecution of pathologists during the Nazi Era (Sziranyi et al. 2019). However, when looking at the emigration patterns of all Jews in Germany, it becomes clear that the majority of Jews did not emigrate until 1939 (Buggle et al. 2023)—a time when most of the pharmacologists analysed here had already emigrated (Löffelholz and Trendelenburg 2008). The reason for the difference between the persecuted pharmacologists and the Jews in Germany as a whole can be found in specific political measures.

On 1 April 1933 there was a so-called ‘Boycott of Jewish Businesses’, in which the Nazis called for the avoidance of Jewish-owned businesses (Medawar and Pyke 2001; Kröner 1989). Jewish lawyers and doctors were the main targets of this anti-Semitic campaign (Beddies et al. 2014). A week later, the ‘Law for the Restoration of the Professional Civil Service’ came into force, leading to a ban on Jewish civil servants and those critical of the regime (Gesetz zur Wiederherstellung des Berufsbeamtentums vom 7. April 1933, Reichsgesetzblatt 1933, Teil I, 51). This was followed by a wave of dismissals of ‘non-Aryan’ doctors from state health institutions, universities and, shortly afterwards, non-civil servants and workers (Beddies et al. 2014; Sziranyi et al. 2019; Gerstengarbe 1994). On 22 April 1933, the licence to practise as a statutory health insurance doctor was withdrawn from 'non-Aryan' and registered doctors and those critical of the regime (Beddies et al. 2014; Kröner 1989).

Finally, in 1938, the licences of Jewish doctors were revoked, leading to the ‘[…] end of the Jewish medical profession’ (Beddies et al. 2014, p. 53). The continued intensification of anti-Semitic measures by the Nazis led to a further wave of emigration of Jewish doctors in that year, including the pharmacologists analysed here (see Fig. 4) (Sziranyi et al. 2019; Kröner 1989; Beddies et al. 2014).

#### Countries of emigration

**Fig. 5:**
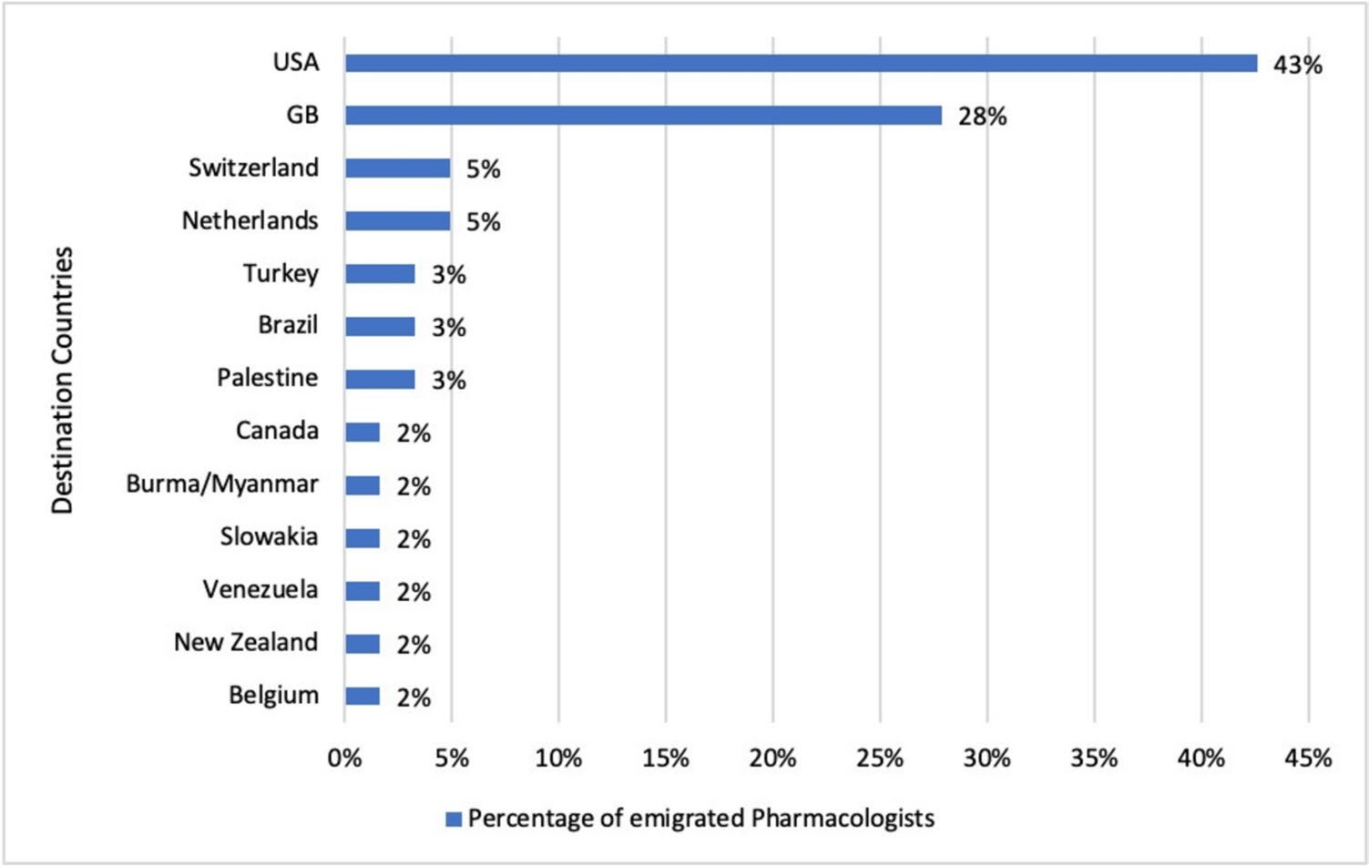
Destination countries of emigrated pharmacologists

We analysed the countries of emigration. The most common destination was the USA, where 43% of the persecuted pharmacologists emigrated (see Fig. 5) (Löffelholz and Trendelenburg 2008). A study analysing the persecution of pathologists in Nazi era found a similar percentage distribution of destination countries of emigration (Sziranyi et al. 2019). The USA was also the most common destination for persecuted dermatologists who emigrated (Eppinger et al. 2003). However, most pharmacologists who emigrated to the USA could not continue their academic careers as hoped (Löffelholz and Trendelenburg 2008; Löffelholz 2011). One notable exception was Otto Krayer (**33** in Table 1), who went on to become Director of the Department of Pharmacology at Harvard Medical School. At the end of his time there, the department ranked high on the American Council on Education’s list of pharmacology departments in the US, and his name is still well known today (Rubin 2014).

The second most popular country for emigrating pharmacologists was Great Britain. There, 28% of persecuted pharmacologists emigrated and were able to continue their scientific achievements at a similar level as before in Germany (Löffelholz and Trendelenburg 2008). In 1933, in response to the dismissal of ‘non-Aryan’ academics from German universities, the charity Academic Assistance Council (AAC) was set up in Great Britain to support discriminated and expelled scientists (Rall 2017). In the 1930s and 1940s, they supported around two thousand outstanding expelled scientists, 20 of whom went on to win Nobel Prizes and 54 of whom were elected Fellows of the Royal Society—including Marthe Vogt (**65** in Table 1), one of the persecuted pharmacologists analysed here (Medawar and Pyke 2001; Rall 2017). 29% of the pharmacologists analysed in this study who emigrated to Great Britain were elected to the British *Pharmacological Society’s Pharmacology Hall of Fame* (https://www.bps.ac.uk/about/about-pharmacology/pharmacology-hall-of-fame) (see Table 2). These developments underscore, on the one hand, the great losses suffered by German science due to the emigration of outstanding scientists and, on the other hand, the enormous gain for British science (Medawar and Pyke 2001; Kohn 2011).

**Table 2:** Persecuted Pharmacologists analysed in this study who are Members of the British Pharmacological Society's Pharmacology Hall of Fame (https://www.bps.ac.uk/about/about-pharmacology/pharmacology-hall-of-fame)

Assigned numbers based on Table 1	Members of the British Pharmacological Society’s Pharmacology Hall of Fame	Year of election
6	Bülbring, Edith	2013
32	Kosterlitz, Hans	2014
38	Loewi, Otto	2016
56	Schild, Heinz Otto	2014
65	Vogt, Marthe Louise	2014

Other destination countries were Switzerland and the Netherlands with 5% each, and Turkey, Brazil and Palestine with 3% each. Canada, Burma/Myanmar, Slovenia, Venezuela, New Zealand and Belgium each accounted for 2% of persecuted pharmacologists who emigrated (see Fig. 5).

The extent to which pharmacologists have enriched the sciences in the countries to which they emigrated is evident from their biographies, awards and honours. Or as Walter Cook, director of the Institute of Fine Arts at New York University, joked: ‘Hitler is my best friend. He shakes the tree and I collect the apples’ (Walter Cook, quoted in Panofsky 1954, S. 332).

#### Metadata on publications—*Naunyn–Schmiedeberg’s Archives of Pharmacology*

To analyse how the dynamics of pharmacologists' publication behaviour changed under persecution, we looked at the total number of publications in *Naunyn–Schmiedeberg's Archives of Pharmacology* and made comparisons with those of the 71 persecuted pharmacologists.

**Fig. 6:**
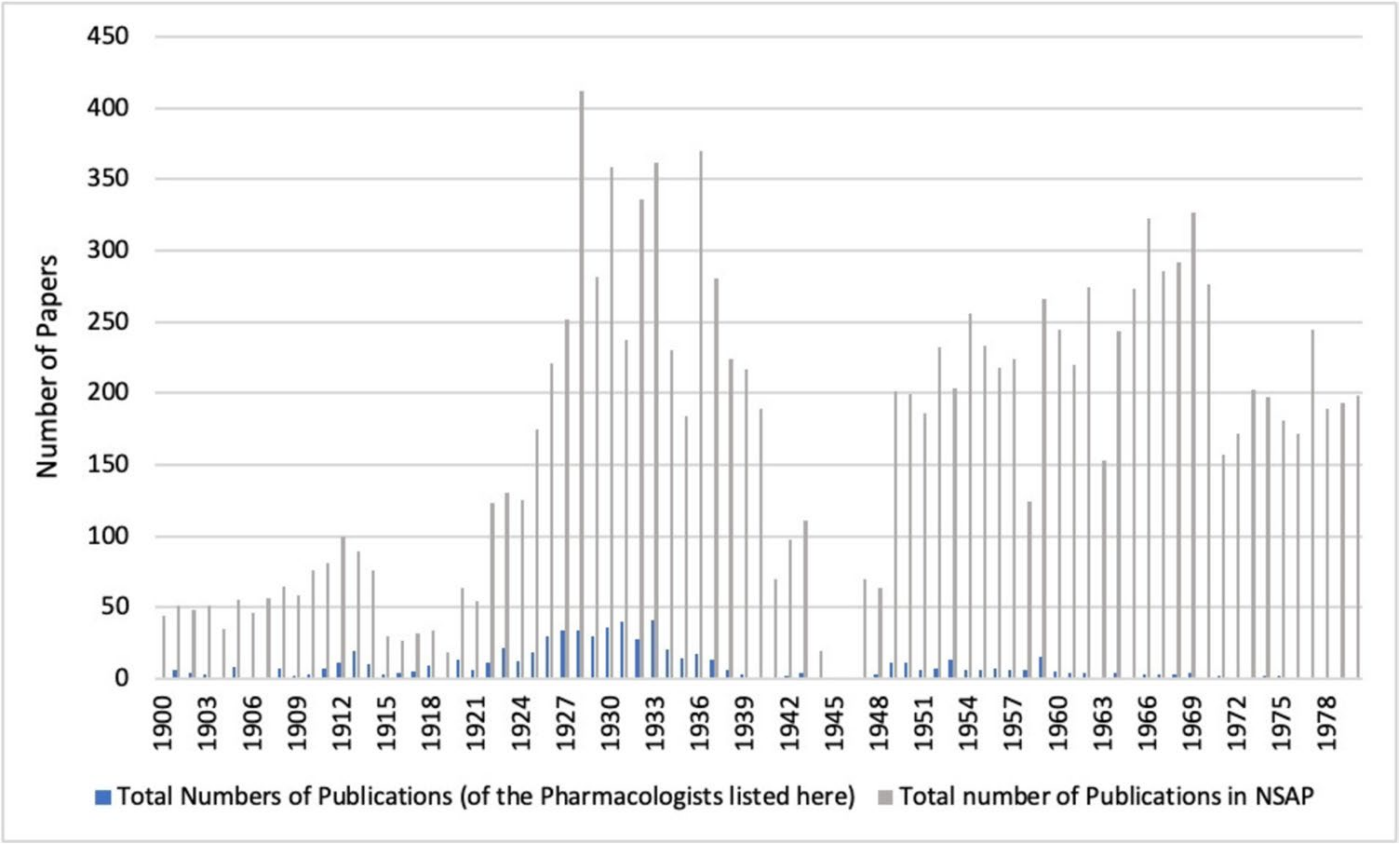
Number of publications (all types of papers) in *Naunyn–Schmiedeberg’s Archives of Pharmacology* (NSAP) per year (absolute numbers)

From the mid-1920s, after the end of the First World War, *Naunyn–Schmiedeberg's Archives of Pharmacology* experienced a rise in the total number of publications (all types of papers), and in the following years this rate stabilized at an average of more than 250 publications per year (see Fig. 6). A strong downward trend can be observed from 1937 onwards, and in 1945/46 there was no publication at all due to the war (publication pause) (Starke 1998). From 1949 onwards, the number of publications increased significantly and remained relatively stable with 200-250 items per year (see Fig. 6).

**Fig. 7:**
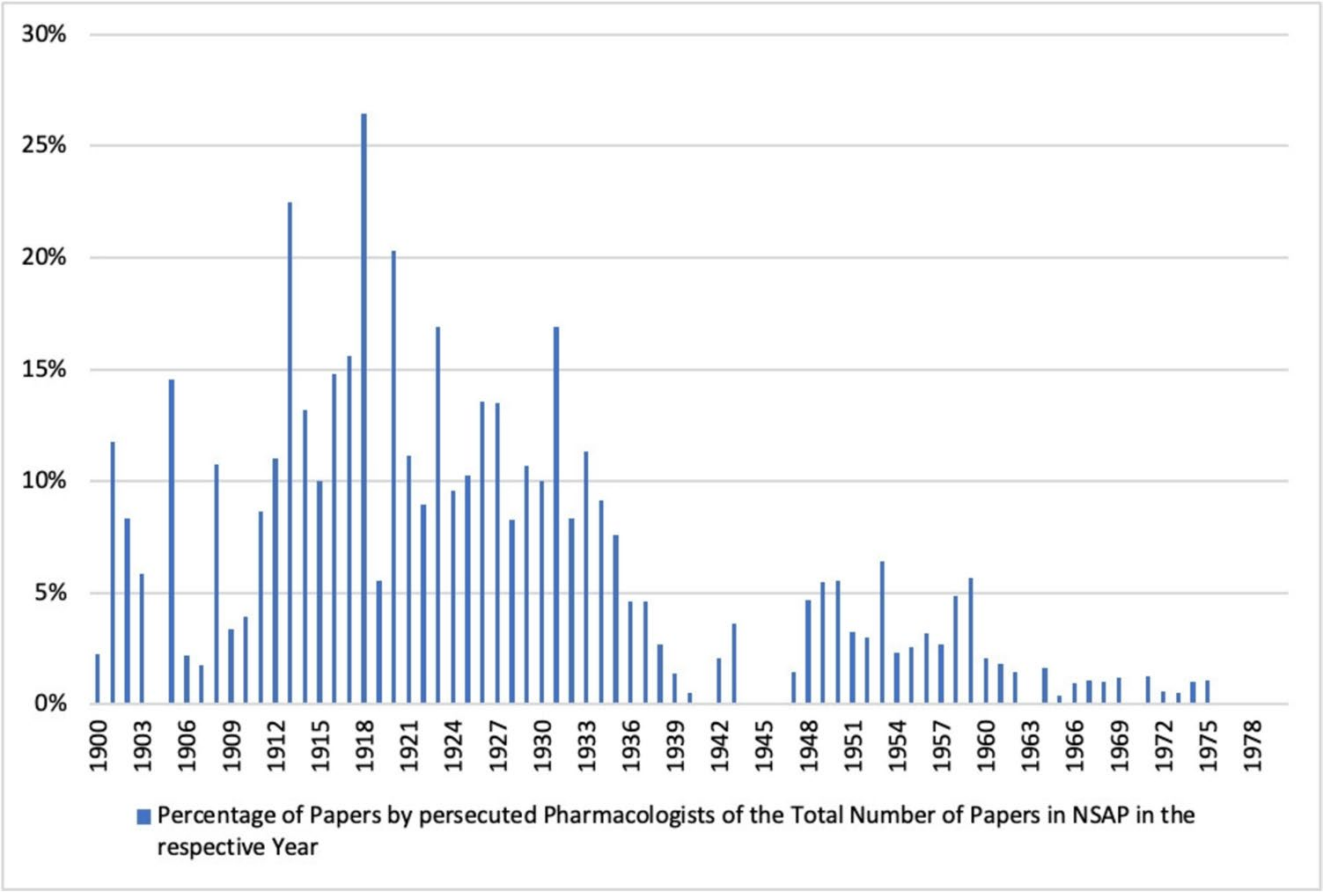
Percentage of papers by persecuted pharmacologists in the total number of papers in *Naunyn–Schmiedeberg’s Archives of Pharmacology* (NSAP) in the respective year

**Fig. 8:**
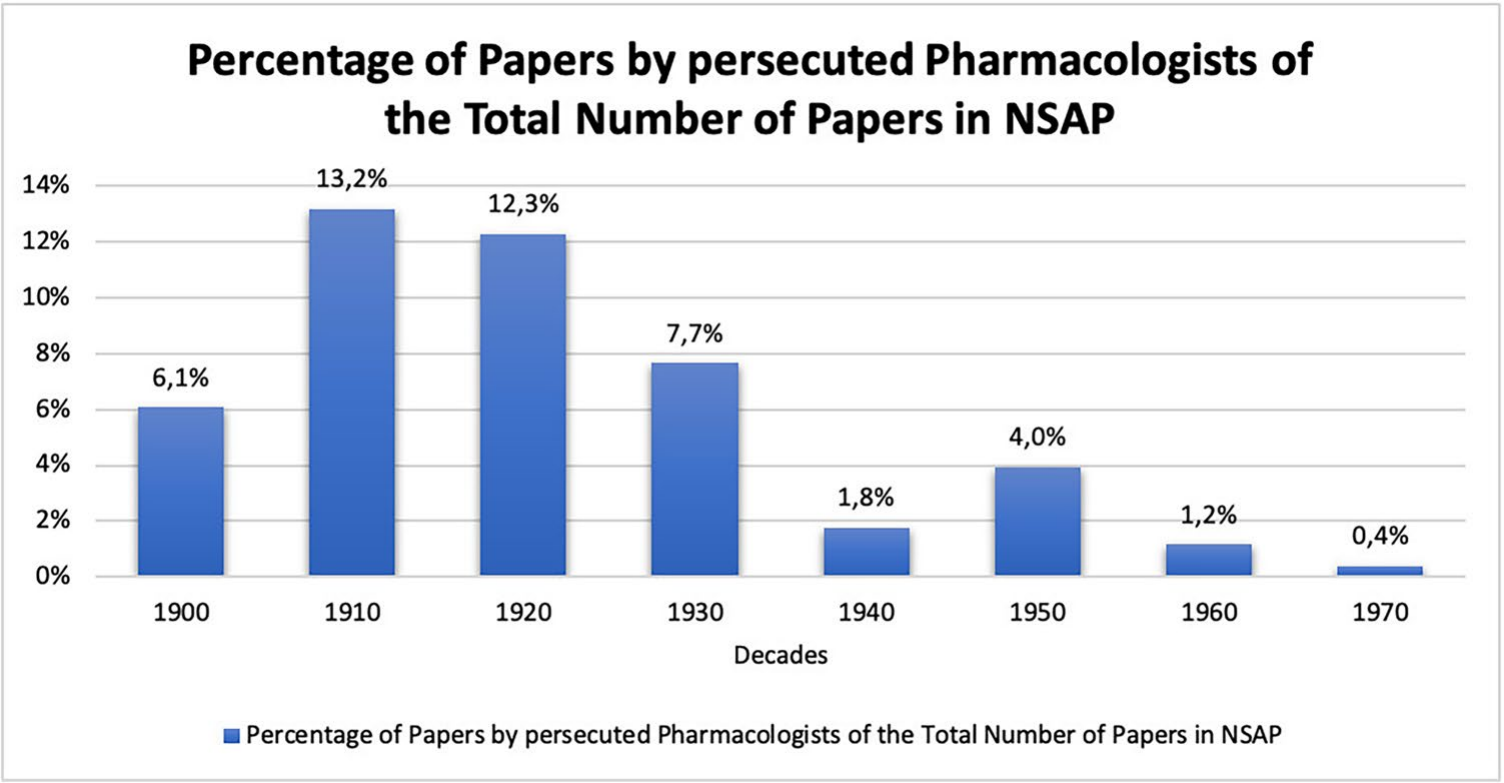
Percentage of papers by persecuted pharmacologists in total number of papers in *Naunyn–Schmiedeberg’s Archives of Pharmacology* (NSAP) in the respective decade

**Fig. 9:**
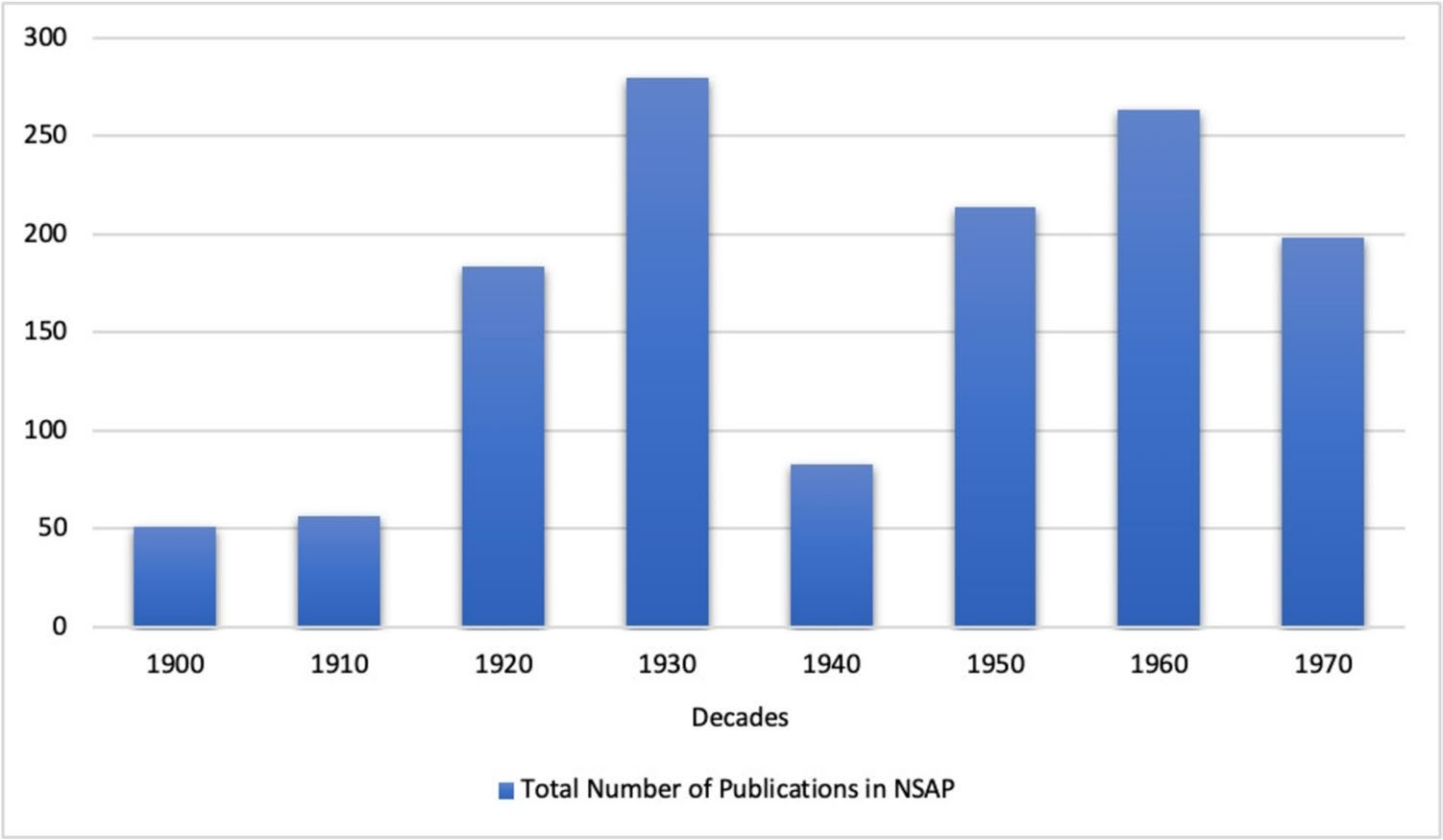
Total numbers of publications in *Naunyn–Schmiedeberg’s Archives of Pharmacology* (NSAP)

Looking at the publications of persecuted pharmacologists, we found that at the beginning of the twentieth century they accounted for an average of 6% of the total publications in *Naunyn–Schmiedeberg’s Archives of Pharmacology*. This proportion doubled in the following two decades, reaching a peak of 13% in the decade from 1910 to 1919 and 12% in the decade from 1920 to 1929. Thus, just 71 pharmacologists contributed to 12% of the research papers published in *Naunyn–Schmiedeberg’s Archives of Pharmacology* in the decade prior to the Nazi era. In the period from 1930 to 1939, which includes the rise to power of the National Socialists and the beginning of the Second World War, there is a sharp decline in their share of total publications. This negative trend was not reversed in the post-war decades; on the contrary, a low of just under 2% was reached in the decade from 1940 to 1949. In the following decades, from 1950 to 1979, the proportion of publications by persecuted pharmacologists in *Naunyn–Schmiedeberg’s Archives of Pharmacology* continued to stagnate between 0–4% (see Figs. 7 and 8). This development is in clear contrast to the development of the overall publications in *Naunyn–Schmiedeberg’s Archives of Pharmacology* (Figs. 9 and 6). After the drastic decline in publications during and in the first years after the Second World War, the journal experienced an upswing due to the introduction of English as the obligatory publication language in the 1970s (Starke 1998).

It should be noted that pharmacology is an interdisciplinary field receiving input from pathology, physiology, microbiology, physiology and biochemistry. This interdisciplinary nature of pharmacology is very well reflected in the first decades of the history of *Naunyn–Schmiedeberg’s Archives of Pharmacology* (Starke 1998). Thus, it is very likely that persecuted scientists from other scientific fields than pharmacology contributed substantially to *Naunyn–Schmiedeberg’s Archives of Pharmacology* as well, but in this study, we did not research these individuals. A systematic analysis of all persecuted scientists who contributed to *Naunyn–Schmiedeberg’s Archives of Pharmacology* will be the subject of future studies. It is very likely that the percentage contribution of persecuted scientists to the journal will increase significantly. But it is also likely that in such analysis, not all persecuted scientists will be covered due to a lack of biographical data.

#### Metadata on publications—*Naunyn–Schmiedeberg’s Archives of Pharmacology*, *Journal of Pharmacology and Experimental Therapeutics* and the *British Journal of Pharmacology*

After analysing the declining number of publications of the persecuted pharmacologists in *Naunyn–Schmiedeberg’s Archives of Pharmacology*, we asked whether the pharmacologists continued their scientific research in their new locations and published their findings in other pharmacological journals.

 43% of the persecuted pharmacologists emigrated to the USA (see Fig. 5). Therefore, we looked at their publications in the *Journal of Pharmacology and Experimental Therapeutics*, the leading American pharmacological journal. Its entire archive is accessible online via the website https://jpet.aspetjournals.org (last accessed October 6th, 2024). The *Journal of Pharmacology and Experimental Therapeutics* was founded in 1909 by the *American Society for Pharmacology and Experimental Therapeutics* (ASPET) and continues to be published monthly.

**Fig. 10:**
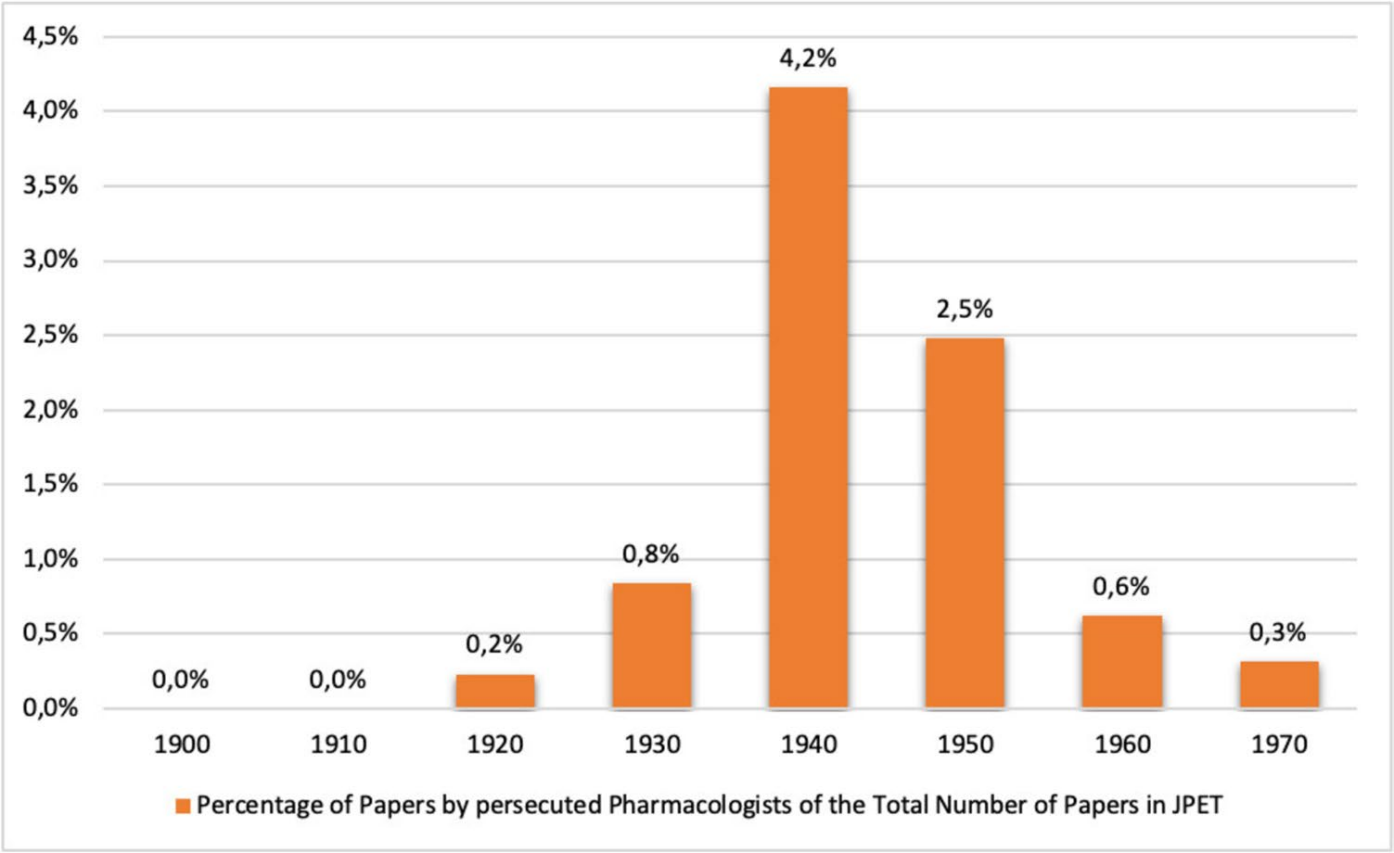
Percentage of papers by persecuted pharmacologists in of the total number of papers in the *Journal of Pharmacology and Experimental Therapeutics* (JPET) in the respective decade

**Fig. 11:**
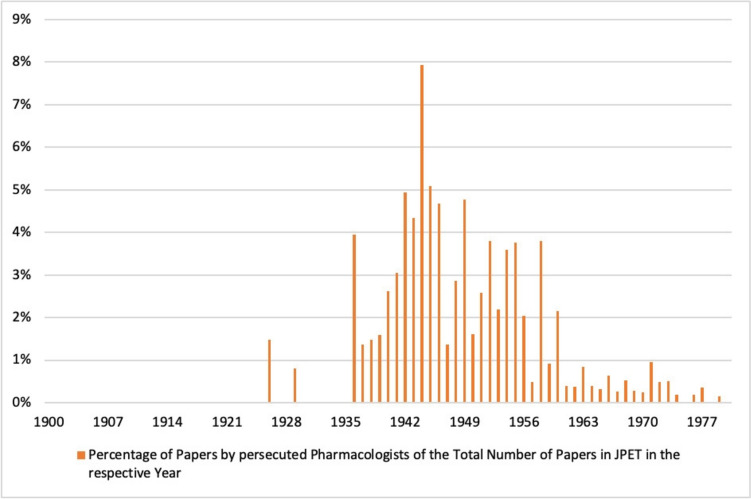
Percentage of papers by persecuted pharmacologists in the total number of papers in the *Journal of Pharmacology and Experimental Therapeutics* (JPET) in the respective year

**Fig. 12:**
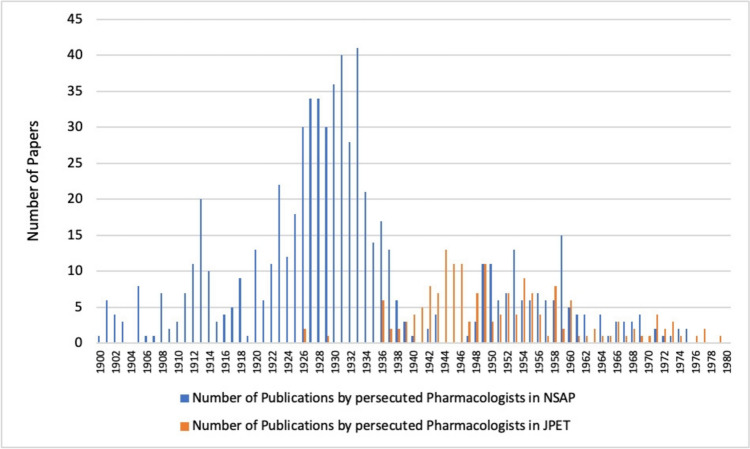
Papers by persecuted pharmacologists in *Naunyn–Schmiedeberg’s Archives of Pharmacology* and *Journal of Pharmacology and Experimental Therapeutics*

Apart from a few isolated publications before 1936, a strong increase in publications by persecuted pharmacologists in the *Journal of Pharmacology and Experimental Therapeutics* can be observed from 1936 onwards. Although the proportion of publications by persecuted pharmacologists from Germany is in the low percentage range, the number of publications increased significantly until 1944, so that their proportion of publications in the *Journal of Pharmacology and Experimental Therapeutics* more than quintupled in the decade from 1930 to 1940 (see Figs. 10 and 11). This corresponds to the period when the majority of pharmacologists persecuted in Germany emigrated (see Fig. 4). Thus, in the journal chosen here, the number of publications by persecuted pharmacologists increased significantly after they emigrated (Figs. 10 and 11), while the number of publications by these pharmacologists in the German journal *Naunyn–Schmiedeberg's Archives of Pharmacology* declined (see Fig. 12).

The next most common destination was Great Britain (see Fig. 5). 28% of the persecuted pharmacologists analysed here emigrated to Great Britain, where they were able to continue and develop their scientific careers (Löffelholz and Trendelenburg 2008). Until the establishment of a British pharmacological journal, the American *Journal of Pharmacology and Experimental Therapeutics* was an important publication venue for British pharmacologists ‘[…] but the anticipated expansion in pharmacological research following the war [World War II] now justified the founding of a new journal devoted to pharmacology’ (Birmingham 1996). The *British Society of Pharmacology* published the first issue of the *British Journal of Pharmacology* in 1946 (Birmingham 1996; Cuthbert 2006).

We examined the scientific contributions of the pharmacologists followed in this journal and compared them with the two previously analysed journals, *Naunyn–Schmiedeberg’s Archive of Pharmacology* and the *Journal of Pharmacology and Experimental Therapeutics*. The full archive of the *British Journal of Pharmacology* is available online at https://bpspubs.onlinelibrary.wiley.com/journal/14765381 (last accessed 11 November 2024).

**Fig. 13:**
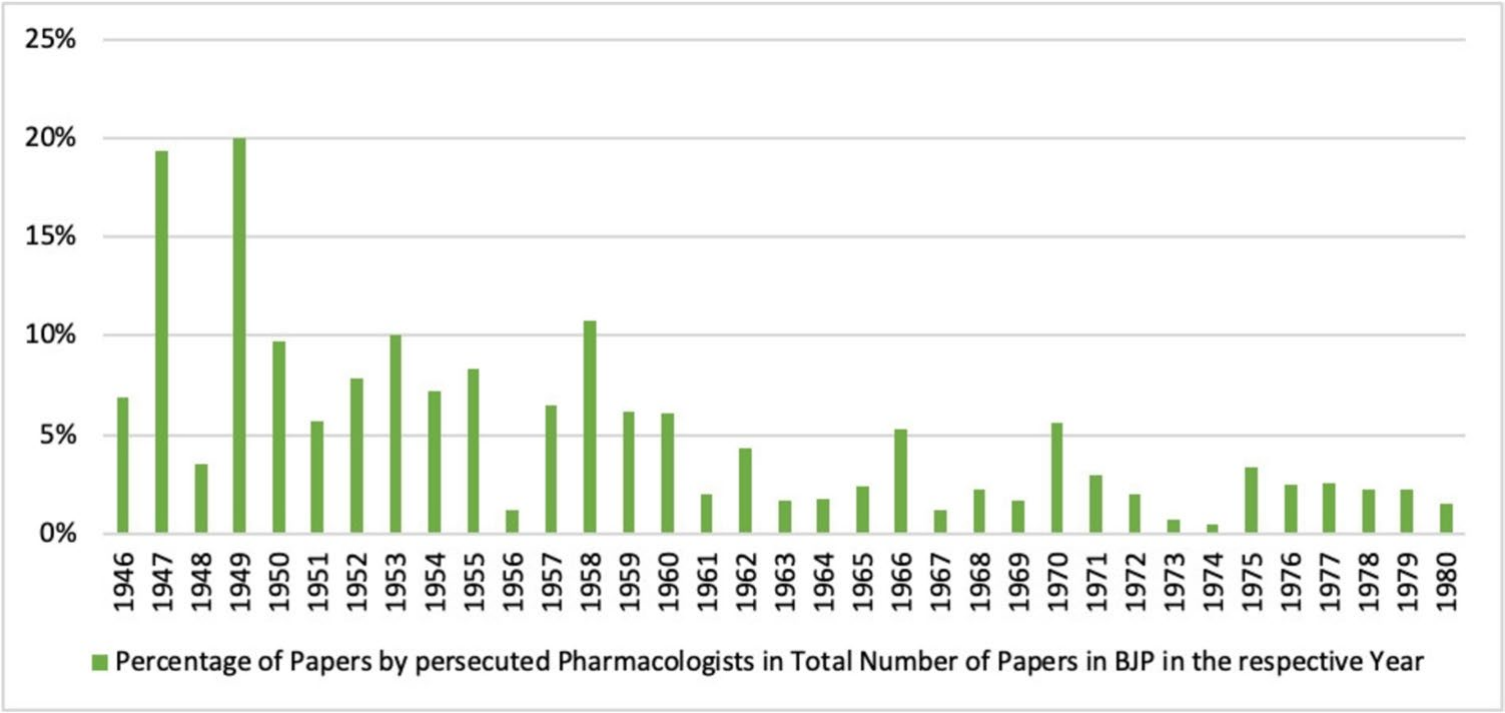
Percentage of papers by persecuted pharmacologists in the total number of papers in the *British Journal of Pharmacology* (BJP) in the respective year

**Fig. 14:**
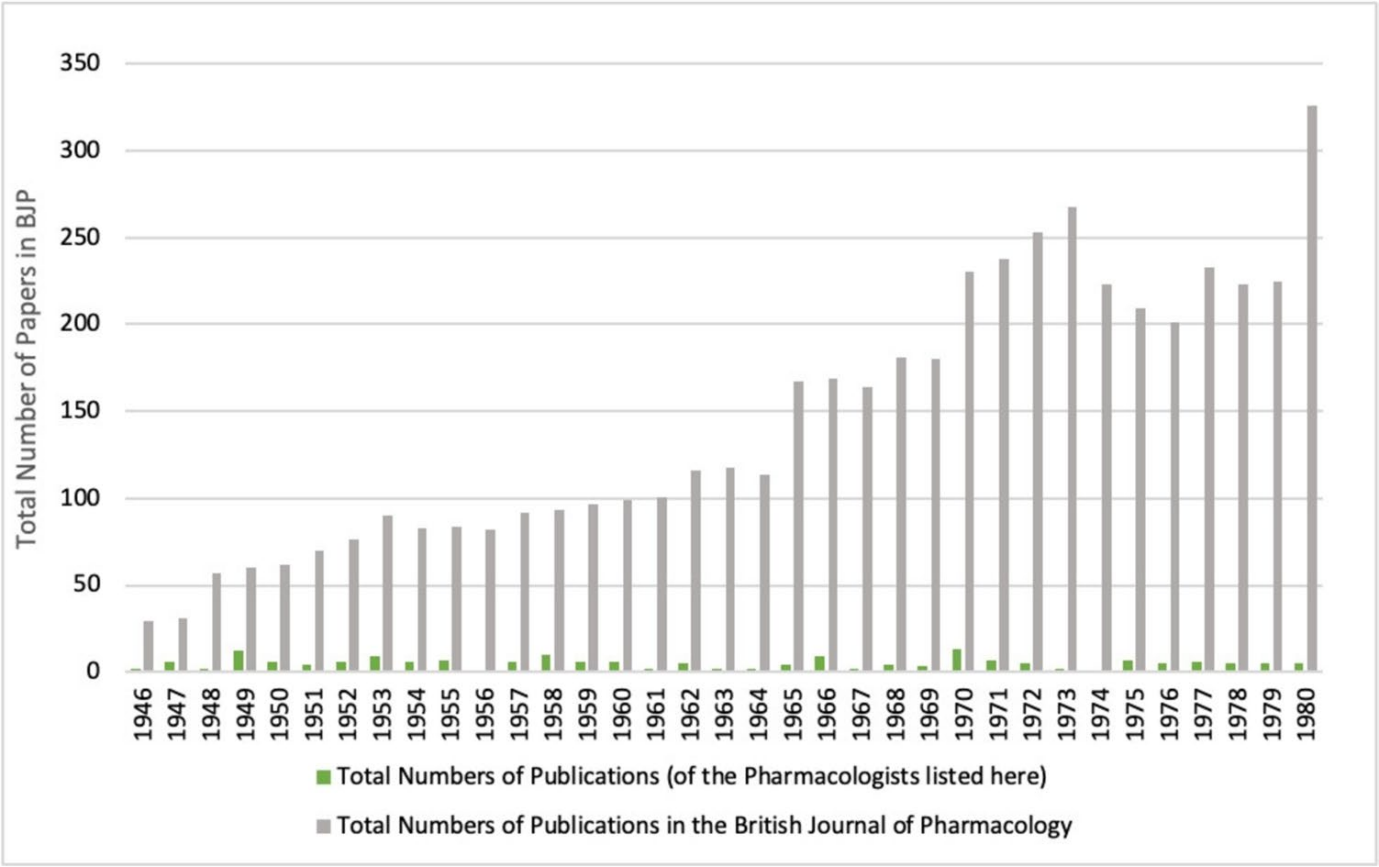
Number of publications in the *British Journal of Pharmacology* (BJP) per year (absolute numbers)

Already in the second year after the foundation of the *British Journal of Pharmacology*, the proportion of publications by persecuted pharmacologists in the *British Journal of Pharmacology* amounted to approximately 20% of the total publications (see Fig. 13). Thus, a comparatively small group of pharmacologists who were persecuted in Germany and emigrated to Great Britain had a considerable influence on the successful development of the new journal. The total number of publications in the *British Journal of Pharmacology* increased steadily after its foundation in 1946 (see Fig. 14). The increase in publication frequency from quarterly to twice monthly since 1995 reflects this development (Birmingham 1996). The increase in the total number of publications led to a decrease in the percentage of publications by the persecuted pharmacologists studied, although their absolute number remained fairly constant over the period analysed (see Fig. 14).

**Fig. 15:**
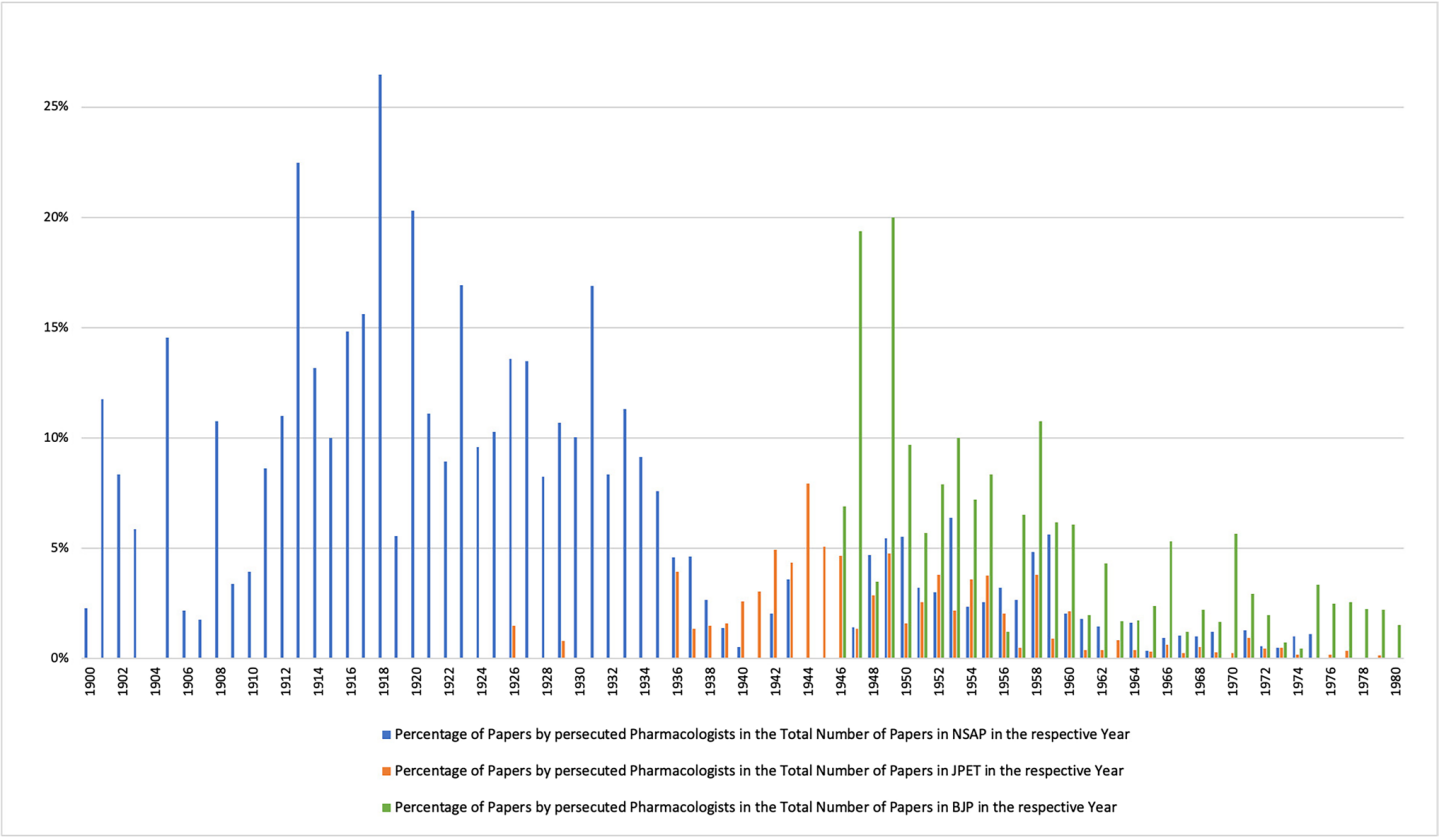
Papers by persecuted pharmacologists in *Naunyn–Schmiedeberg’s Archives of Pharmacology*, *Journal of Pharmacology and Experimental Therapeutics* and *British Journal of Pharmacology*

Figure 15 directly compares *Naunyn–Schmiedeberg’s Archives of Pharmacology, American Journal of Pharmacology and Experimental Therapeutics* and *British Journal of Pharmacology*. It impressively reveals that the persecuted German pharmacologists largely abandoned *Naunyn–Schmiedeberg’s Archives of Pharmacology* as primary target journal after emigration in favour of the *Journal of Pharmacology and Experimental Therapeutics* and (after World War II) the *British Journal of Pharmacology*. Among the pharmacologists who emigrated were eminent scientists such as Otto Krayer, Marthe Louise Vogt, Otto Loewi (Nobel Laureate) and Wilhelm Siegmund Feldberg (see **33**, **65**, **38** and **11** in Table 1), whose important contributions to the German journal were thus lost. Figure 15 also impressively documents that after the disruption of the scientific work because of the persecution, leading to a low in publications between 1936–1940, the persecuted pharmacologists strongly recovered in productivity and made important scientific contributions to pharmacology for the next decades. This extraordinary achievement of the persecuted pharmacologists under most difficult personal and political conditions documents their enormous talent, stamina and dedication to pharmacology. The impressive number of publications shown in Fig. 15 must also be seen under the aspect that numerous persecuted pharmacologists had no or very little professional opportunities for publications after 1933. In other words, Fig. 15 does not simply reflect a switch of target journals but also a huge loss of research that could not be conducted and papers that could not be published.

The damage to the impact, reputation and international standing of *Naunyn–Schmiedeberg’s Archives of Pharmacology* inflicted by the Nazi regime was long-lasting and was reverted only decades later (Starke 1998; Dats et al. 2023).

Since pharmacology is interdisciplinary, it is very likely that persecuted pharmacologists also published in physiology, microbiology, biochemistry and clinical journals. These additional target journals were not covered here for the sake of focus. A systematic analysis of all journals in which persecuted pharmacologists published, will be the focus of future studies.

#### Metadata topics

**Fig. 16:**
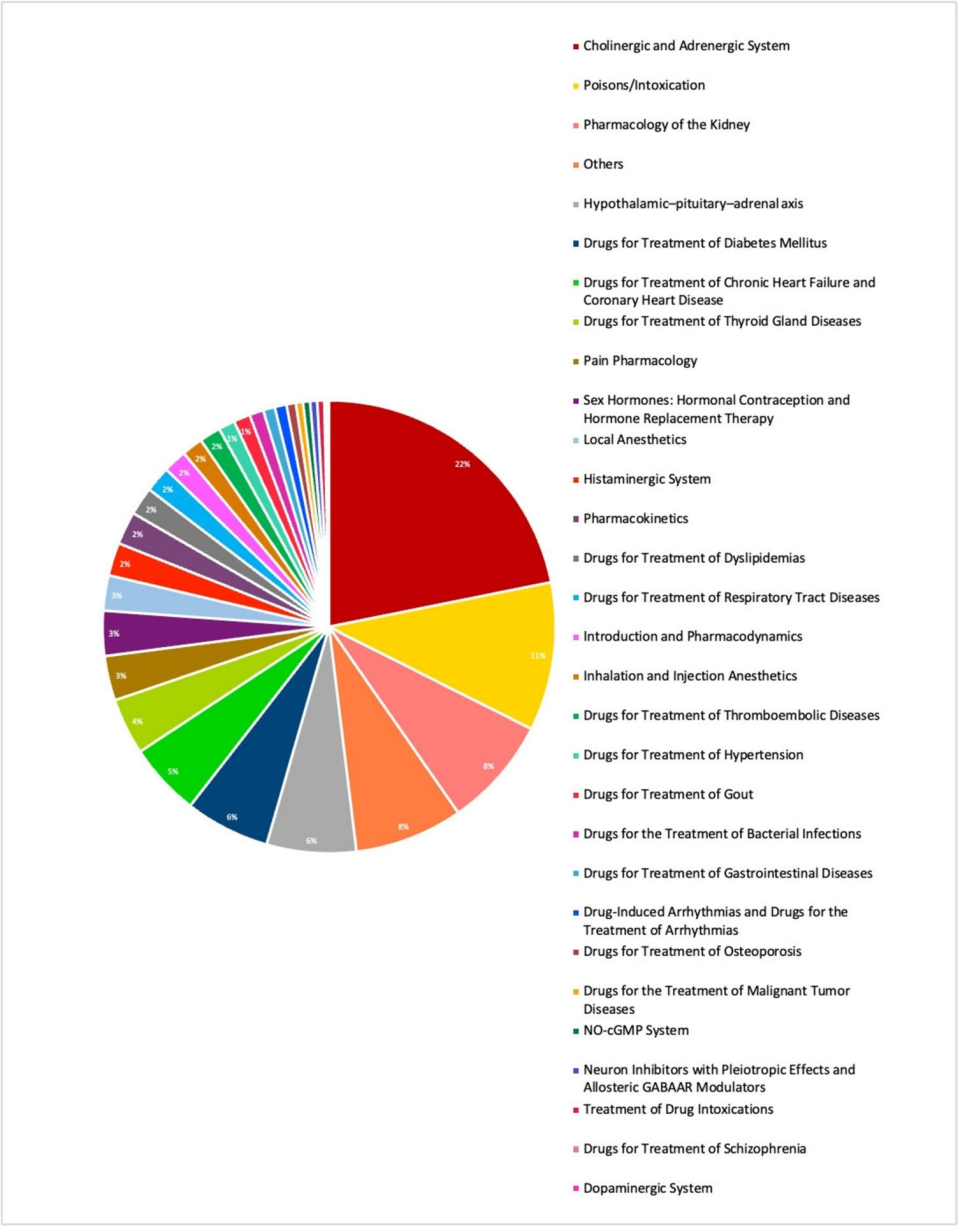
Topics of Publications in *Naunyn–Schmiedeberg’s Archives of Pharmacology* (NSAP) by persecuted pharmacologists between 1900 and 1980

We assigned the publications of persecuted pharmacologists from 1900 to 1980 to one chapter each of the textbook *‘Basic Knowledge of Pharmacology*’ (Seifert 2019) and added the topics ‘Poisons and Intoxication’ and ‘Hypothalamic–Pituitary–Adrenal-Axis’. The most common topic was ‘Cholinergic and Adrenergic System’ (22%), followed by ‘Poisons and Intoxications’ (11%). 8% of the papers could be assigned to the topic ‘Pharmacology of the Kidney’, while the topics ‘Hypothalamic–Pituitary–Adrenal Axis’ and ‘Drugs for Treatment of Diabetes Mellitus’ accounted for 6%. 5% of the papers were on ‘Drugs for Treatment of Chronic Heart Failure and Coronary Heart Disease’ and 4% of the papers were on ‘Drugs for Treatment of Thyroid Gland Diseases’. All other main topics in the textbook could be assigned to ≤ 3% of the papers (Fig. 16).

**Fig. 17:**
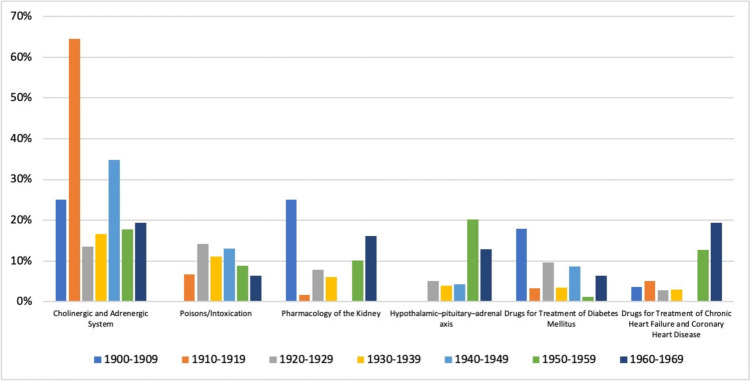
Development of the six most frequent topics of the papers of persecuted pharmacologists in *Naunyn–Schmiedeberg’s Archives of Pharmacology* (NSAP) in decades over the period 1900–1969. Percentages indicate the respective topic’s share in the total number of publications in all topics in each decade

We also analysed the development of the six most common research topics from 1900 to 1970. The ‘Cholinergic and Adrenergic System’ became the dominant research topic in the 1910s, accounting for more than 60% of the published papers, with a further peak in the 1940s. ‘Poisons and Intoxication’ remained constant over the decades, always accounting for about 10% of all publications. The topic ‘Pharmacology of the Kidney’ peaked in the early 1900s, followed by a resurgence in the 1950s and 1960s. The ‘Hypothalamic–Pituitary–Adrenal Axis’ was also mainly researched in the 1950s and 1960s. ‘Drugs for Treatment for Diabetes Mellitus’ attracted a great deal of attention, especially in the early 1900s, while research into ‘Drugs for Treatment of Chronic Heart Failure and Coronary Heart Disease’ became increasingly important in the 1950s and 1960s (Fig. 17).

The analysis shows the great variability and diversity of topics in pharmacological research by persecuted German pharmacologists. It is noteworthy that the topics we have added, ‘Poisons and Intoxication’ and ‘Hypothalamic–Pituitary–Adrenal Axis’, were among the most common topics. This underscores the close relationship of the ‘sister sciences’ pharmacology and toxicology, and the origin of pharmacology from physiology and its close historical connection (Dollery 2006; Starke 1998).

When the research topics of the persecuted pharmacologists are compared with the research topics in *Naunyn–Schmiedeberg’s Archives of Pharmacology* after World War II, it is noted that for the journal in its entirety, the three topics pharmacodynamics, pharmacokinetics and cholinergic/adrenergic system are very dominant (Dats et al. 2023; Basol and Seifert 2024a, b), whereas among the persecuted pharmacologists, a much broader spectrum of research topics is represented, diverging from the main stream (Figs. 13 and 14). Thus, persecuted pharmacologists covered many areas of research that were lost to the *Naunyn–Schmiedeberg’s Archives of Pharmacology* as a result of persecution. The broad diversity of research topics presented in *Naunyn–Schmiedeberg’s Archives of Pharmacology* from 1873–1933 was not re-instated until 2010–2024 (Dats et al. 2023), impressively demonstrating that the persecution of pharmacologists in Germany had a long-lasting negative effect on the topics covered in *Naunyn–Schmiedeberg’s Archives of Pharmacology* for decades. Due to the focus on few topics, the journal was perceived as a rather specialized “neurotransmitter journal” for decades (Dats et al. 2023) although the instructions for authors clearly state that the journal covers all fields of pharmacology. While the journal survived after World War II with this focus, it prevented *Naunyn–Schmiedeberg’s Archives of Pharmacolog*y from nurturing other fields of pharmacology, thereby making it very vulnerable to being marginalized (Starke 1998; Dats et al. 2023; Basol and Seifert 2024a, b). The extinction of *Naunyn–Schmiedeberg’s Archives of Pharmacology* was only prevented by rigorous internationalization, introducing English as mandatory language and opening the journal to new scientific topics and emerging countries such as China, India, Iran, Egypt, Turkey and Brazil (Starke 1998; Dats et al 2023). Taken together, the analysis of the research topics of persecuted pharmacologists revealed that they represented a huge spectrum of scientific expertise that was lost to German pharmacology for decades but was effectively integrated into their emigration countries.

#### Metadata of articles published between 1933 and 1945

As part of our research, we analysed the papers of persecuted pharmacologists published in *Naunyn–Schmiedeberg’s Archives of Pharmacology* between 1933 and 1945. During this period, only a few articles by persecuted pharmacologists were published in *Naunyn–Schmiedeberg’s Archives of Pharmacology* (see Figs. 6 and 7). We analysed these contributions in more detail to understand how publications were possible during a period of severe repression of Jewish and dissident scientists (Beddies et al. 2014; Gerstengarbe 1994).

**Fig. 18:**
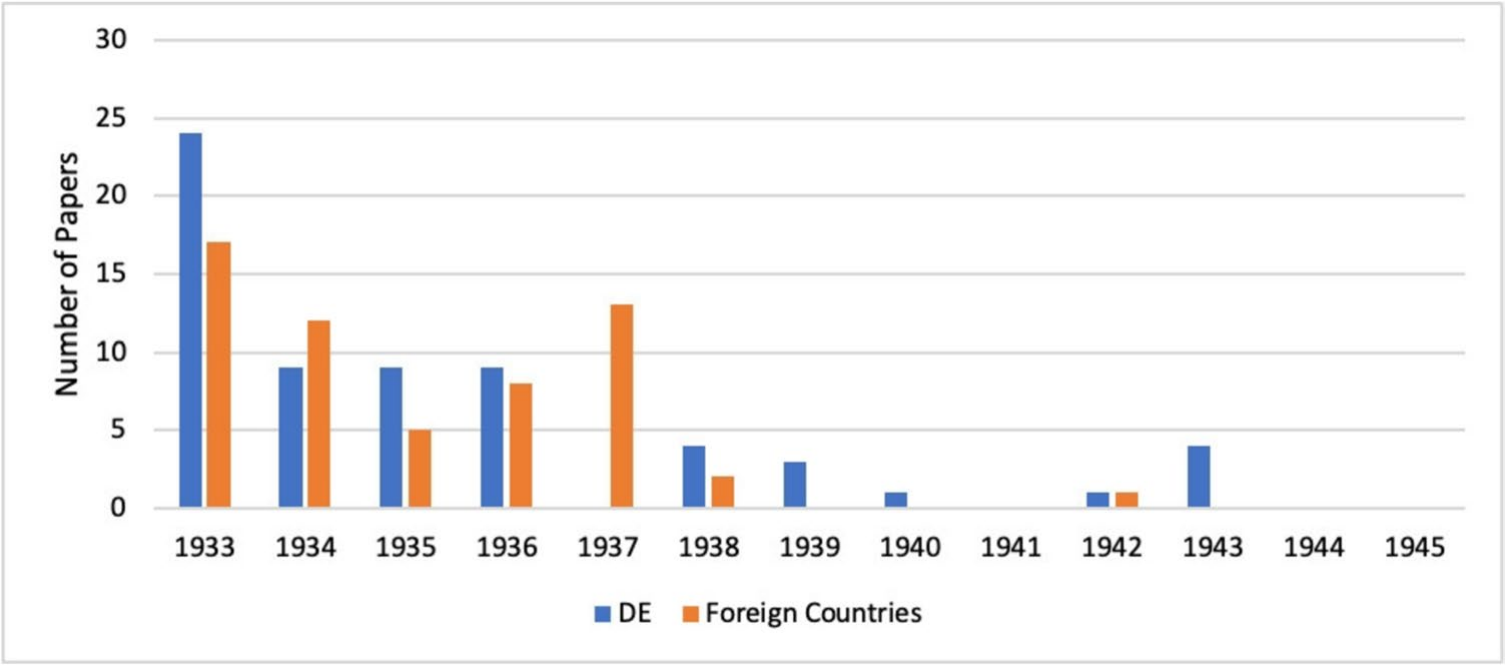
Papers published by persecuted pharmacologists in *Naunyn–Schmiedeberg’s Archives of Pharmacology* between 1933 and 1945. DE, Deutschland (Germany)

We analysed the origin of the published papers and first assigned them to one of the two categories ‘Germany’ or ‘Abroad’ (see Fig. 18). While 52% of the publications came from German institutes, 48% of the articles were published abroad. The analysis also shows that the majority of the articles published by persecuted pharmacologists in Germany between 1933 and 1945 were already published in 1933, while in the following years fewer and fewer articles were published. The reasons for this were the increasing intensification of the suppression and marginalisation of Jewish and dissident scientists (Beddies et al. 2014), the first wave of dismissals of university lecturers in 1933 (Gerstengarbe 1994) and the fact that many of the persecuted pharmacologists had already emigrated after 1933 (see Fig. 4). Almost all of the papers published by persecuted pharmacologists from abroad were published between 1933 and 1937; only a few papers were published after 1938.

**Fig. 19:**
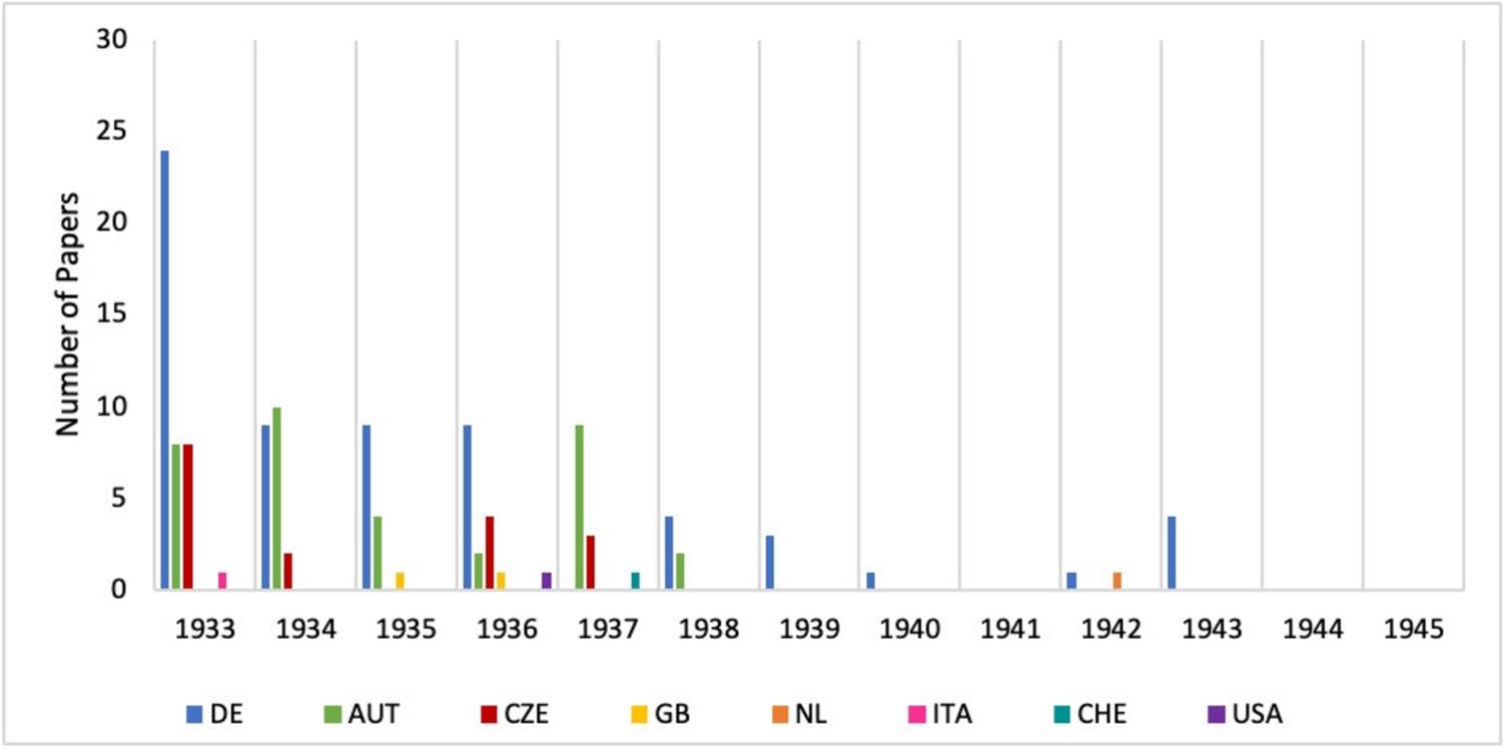
Years and countries of origin of papers published by persecuted pharmacologists in *Naunyn–Schmiedeberg’s Archives of Pharmacology* between 1933 and 1945. DE: Germany, AUT: Austria, CZE: Czechoslovakia, GB: Great Britain, NL: Netherlands, ITA: Italy, CHE: Switzerland, USA: United States of America

The specific countries of origin of the papers published between 1933 and 1945 are shown in Fig. 19. In addition to Germany (52%), these were mainly Austria (29%) and the Czech Republic (14%). Some of the academics first emigrated to neighbouring countries such as Austria or the Czech Republic, as they hoped to return to Germany soon and felt that emigrating overseas was not worthwhile (Löffelholz and Trendelenburg 2008; Medawar and Pyke 2001). From there they continued to publish in *Naunyn–Schmiedeberg’s Archives of Pharmacology*. However, after the annexation of Austria by Germany in 1938, the number of scientific papers submitted from Austria also dried up. The expulsion of Jews was even faster and more aggressive there than in Germany (Friedländer 2007). As the prospect of returning to Germany became increasingly remote after 1938, pharmacologists from Austria and the Czech Republic also fled to more distant countries with better career prospects (Löffelholz and Trendelenburg 2008). This development can be seen in the chart ‘Years and Countries of Origin of Papers published between 1933 and 1945’ (Fig. 19).

But how did the publications of persecuted pharmacologists from German research centres come about? In order to gain an impression of how publications from Germany were possible in the particularly difficult period between 1933 and 1945, we analysed the institutional conditions under which these scientific works were produced.

**Fig. 20:**
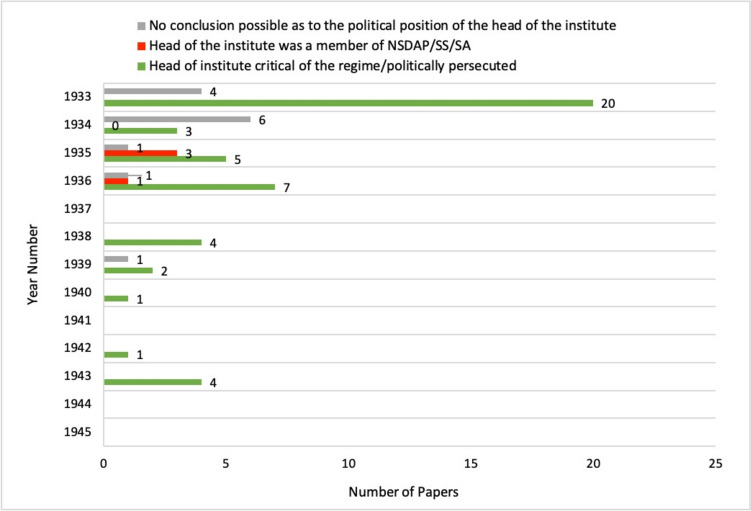
Papers from by persecuted pharmacologists in *Naunyn–Schmiedeberg’s Archives of Pharmacology* Germany between 1933 and 1945—presumed political position of the Institute Directors

We analysed the biographies of the respective institute directors and paid particular attention to the (presumed) political positioning of the institute directors in the context of the Nazi regime. We found that most of the papers published during this period (69%) were by institute directors who were critical of the regime or who were persecuted (see Fig. 20). This suggests that this group of pharmacologists showed solidarity with each other and supported each other to enable persecuted pharmacologists to continue their scientific work in Germany (Medawar and Pyke 2001).

In contrast, only a small proportion (9%) of the papers published by persecuted pharmacologists appeared under institute directors who were documented members of the NSDAP, SS and/or SA. In 22% of the papers analysed, we were unable to draw any conclusions about the political position of the institute directors due to insufficient information about them. An extensive paper analyzing the political positioning of the institute directors dring the Nazi regime, analyzing original archive material, is currently under review in *Naunyn-Schmiedeberg's* *Archives* *of* *Pharmacology.* 

The analysis of the papers published by persecuted pharmacologists between 1933 and 1945 shows on the one hand how severely the scientific work of persecuted pharmacologists was restricted by the Nazi regime, and on the other hand that it was nevertheless possible for a few scientists to work and publish in this hostile environment.

## Conclusions

The expulsion of Jewish and dissident pharmacologists between 1933 and 1945 in Germany had a major long-lasting negative impact on German pharmacology. Using the example of the publication behaviour of persecuted pharmacologists during the Nazi period in Germany in *Naunyn–Schmiedeberg’s Archives of Pharmacology* and the comparable *Journal of Pharmacology and Experimental Therapeutics* and *British Journal of Pharmacology* this study shows how German pharmacology lost much of its publication power because of the expulsion of top-class pharmacologists. The emigration of the scientists analysed here led to a brain drain from which the countries of refuge benefited (see Fig. 12) (Kohn 2011; Rall 2017; Gerstengarbe 1994; Kröner 1989). The excellence of the expelled pharmacologists is also demonstrated by the fact that 29% of the pharmacologists who emigrated to Great Britain were elected to the Pharmacology Hall of Fame of the British Pharmacological Society and 6% of them were Nobel Prize winners (**38** in Table 1) (Rubin 2007; https://www.bps.ac.uk/about/about-pharmacology/pharmacology-hall-of-fame, last accessed October 14, 2024) (see Table 2).

The great loss to German science due to the expulsion of Jewish and dissident scientists is also illustrated by the fact that until 1932, 25% of Nobel Prize winners were of Jewish origin, whereas only 1% of the German population was Jewish (Rall 2017). The German academic community was severely weakened by their persecution, emigration, deportation and murder (Gerstengarbe 1994; Grüttner and Kinas 2007). Persecuted German pharmacologists represented a very broad spectrum of research topics (see Fig. 13) that were lost for decades to German pharmacology as documented by the development of research topics published in *Naunyn–Schmiedeberg’s Archives of Pharmacology*. The re-instatement of the original thematic diversity of the journal has not been accomplished until recently due to continued efforts of the Editors-in-Chief of the past 75 years (Dats et al. 2023 and https://link.springer.com/journal/210, last accessed October 13, 2024).

Specific political events influenced the emigration waves of pharmacologists around 1933 and 1938 (see Fig. 4) (Löffelholz and Trendelenburg 2008), such as the ‘Boycott of Jewish Businesses’ and the entry into force of the ‘Law for the Restoration of the Professional Civil Service’ in April 1933, as well as the withdrawal of licences in 1938 (Beddies et al. 2014; Gerstengarbe 1994; Grüttner and Kinas 2007).

The results of this study contribute to the reappraisal of history and the recognition of the scientific achievements of the persecuted pharmacologists. Unfortunately, this study on the history of Germany is currently becoming sadly topical. It should serve as a reminder of the importance of learning from history and ensuring the protection of scientific integrity as well as the rights and dignity of all scientists (like of all human beings), regardless of their origin or ethnicity.

Our study shows how political ideologies can have a far-reaching negative impact on scientific freedom and progress. This was true not only for pharmacology across Germany and Austria between 1933 and 1945 but also for pharmacology in East-Germany and East-Berlin in the decades following World War II (Basol and Seifert 2024a,b). In contrast, the positive impact of scientific freedom is impressively documented for the dynamic development of pharmacology in West-Berlin in the period from 1947 to 1974, culminating into the formation of the highly successful ‘Günter Schultz school of pharmacology’ (Basol and Seifert 2024a).

In a study that is currently underway and goes far beyond the scope of this global study, we analyse the individual scientific contributions and achievement of the persecuted pharmacologists listed in Table 1 to make them and their achievements comprehensively visible to the international research community.

### Outlook

It can only be speculated how pharmacology in Germany and *Naunyn–Schmiedeberg’s Archives of Pharmacology* would have developed without the persecution of the distinguished pharmacologists listed in Table 1. But it is safe to assume that the diversity of research topics would have been much broader than it was after World War II and that *Naunyn–Schmiedeberg’s Archives of Pharmacology* would not have struggled for decades for its position and reputation in pharmacology. Notably, we did not consider in this paper how many talented students and postdocs of the persecuted pharmacologists listed in Table 1 were lost for German pharmacology and as authors for *Naunyn–Schmiedeberg’s Archives of Pharmacology*.

This paper is a strong warning that populistic and nationalistic political agendas, disregarding the fundamental principles of science, can have an extremely long-lasting negative impact on the scientific development of a country and that recovery from even relatively short-lasting agendas (in this case study just 12 years) can take decades and huge scientific and moral dedication of the next generations of scientists.

## Data Availability

All source data for this study are available upon reasonable request from the authors.
